# Supplementary material for: Identification of a critical lipid ratio in raft-like phases exposed to nitric oxide: An AFM study
Source: Biophys J. 2021 Jun 29;120(15):3103–11. doi: 10.1016/j.bpj.2021.06.009 (PMC8390956; doi:10.1016/j.bpj.2021.06.009)
Supplement: Document S1. Figs. S1–S5 [file mmc1.pdf]

Biophysical Journal, Volume *120*

Supplemental information

Identification of a critical lipid ratio in raft-like phases exposed to nitric oxide: An AFM study

Sanjai Karanth, Amir Azinfar, Christiane A. Helm, and Mihaela Delcea

Supplementary Information

Identification of a critical lipid ratio in raft-like phases exposed to nitric oxide: An AFM study

Sanjai Karanth[1, 2], Amir Azinfar[3], Christiane A. Helm[3] and Mihaela Delcea[1, 2, 4*]

[1]Institute of Biochemistry, University of Greifswald, Felix-Hausdorff-Straße 4, 17489 Greifswald, Germany;
[2]ZIK-HIKE, Zentrum für Innovationskompetenz "Humorale Immunreaktionen bei kardiovaskulären Erkrankungen", Fleischmannstraße 42, 17489 Greifswald, Germany;
[3]Institute of Physics, University of Greifswald, Felix-Hausdorff-Straße 6, 17489 Greifswald, Germany;
[4]DZHK (Deutsches Zentrum für Herz-Kreislauf-Forschung), partnersite Greifswald, Germany.
**Corresponding author: delceam@uni-greifswald.de*

Figure S1: Force histogram of tip-mica adhesive force to determine the non-specific interactions.

Figure S2: Break-through of raft-like phases with different SM and NO concentrations.

Figure S3. Lipid peroxidation assay to detect formation of MDA using N-palmitoyl-D-erythro-sphingosylphosphorylcholine (100% 16:0 SM, Avanti Polar Lipids, AL, USA).

Figure S4: Pull-out force histogram, AFM images and height profiles of POPC:POPS bilayer treated with nitric oxide.

Figure S5: Pull-out force histogram, AFM images and height profiles of DMPC:DMPG bilayer treated with nitric oxide.

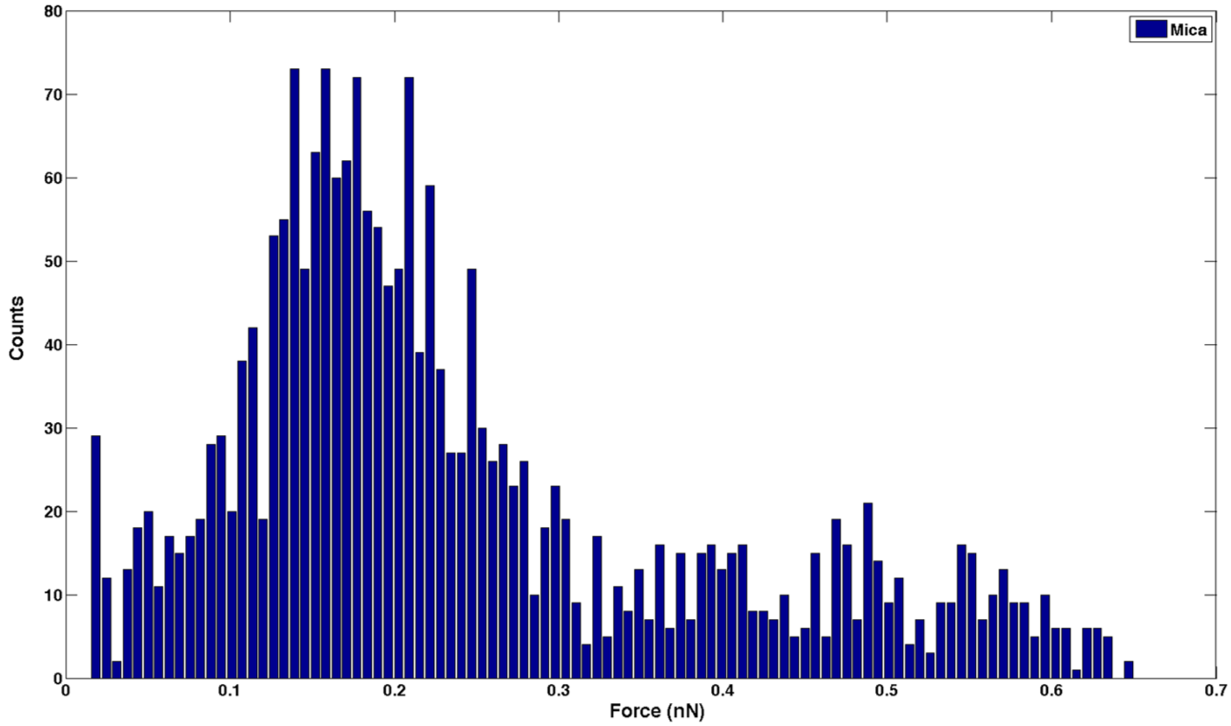

Figure S1: Histogram of pull-out forces between tip and mica surface to determine the non-specific attractive interactions. The histogram displayed forces ranging between 0.02 - 0.65 nN with maximum forces at around 0.2 nN.

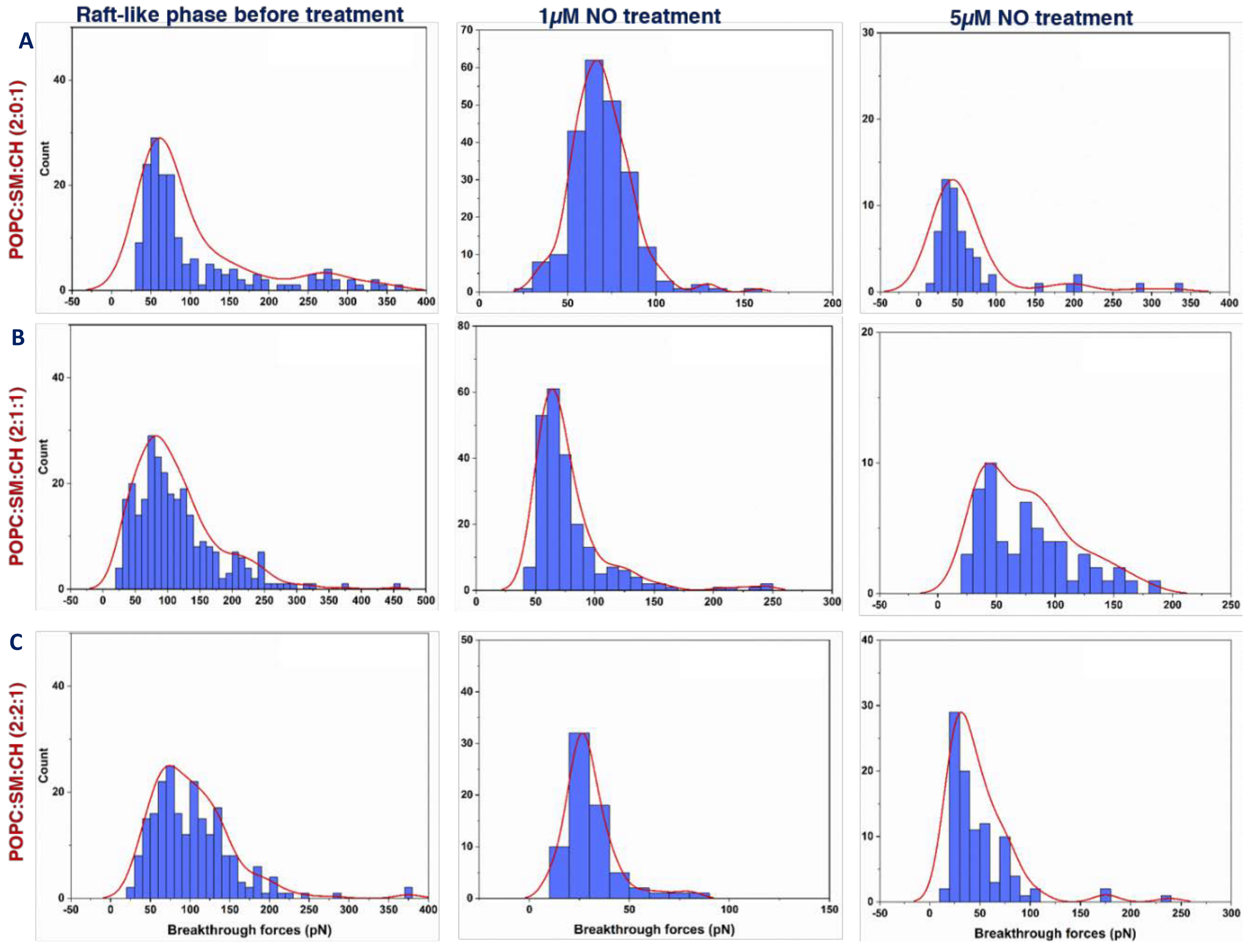

Figure S2: Histograms of break-through forces of raft-like phases (R_LP) with different SM concentration and NO treatment. Control samples *(left column)* showed break-through forces with a large distribution ranging between 25 pN - 400 pN. However, with increase in SM concentration, the distribution of break-through forces became more concentrated with half-width maxima of the Gaussian fit getting broader with SM addition. The peak break-through forces for 2:0:1 was found to be 68 pN *(A, left)* which increased to 80 pN *(B, left)* for the 2:1:1 ratio. Break-through forces were observed at 80 pN and 125 pN *(C, left)*, at almost equal intensities for 2:2:1 ratio. With addition of NO, the break-through forces and their distribution reduced for the 2:0:1 ratio and ranged from 25-150 pN *(A, middle)* without significant change in the peak force. This behavior was similar for 1μM NO (peak force=68 pN) and 5μM NO (peak force=50 pN) concentration. The 2:1:1 ratio showed slight reduction in the peak break-through forces from 80 pN to 75 pN at 1μM NO and 48 pN and 80 pN for 5 μM NO *(B, middle and right)*. A constant breakthrough force of 80 pN was detected throughout. However, the distribution seemed affected regardless of NO concentration with the force range changing from 25 pN -400 pN (control) to 25 pN- 250pN. The 2:2:1 ratio showed drastic changes in the break-through forces with peak force reduced to 27 pN at 1μM NO and 32.5 pN for 5μM NO *(C, middle and right)*. The force distribution was reduced and ranged between 25-100 pN for both NO concentrations. The above obtained forces correlated well with the pull-out forces (Figure 3 in the manuscript) giving a comprehensive understanding on the change in membrane permeability due to SM. Fit = KDE function.

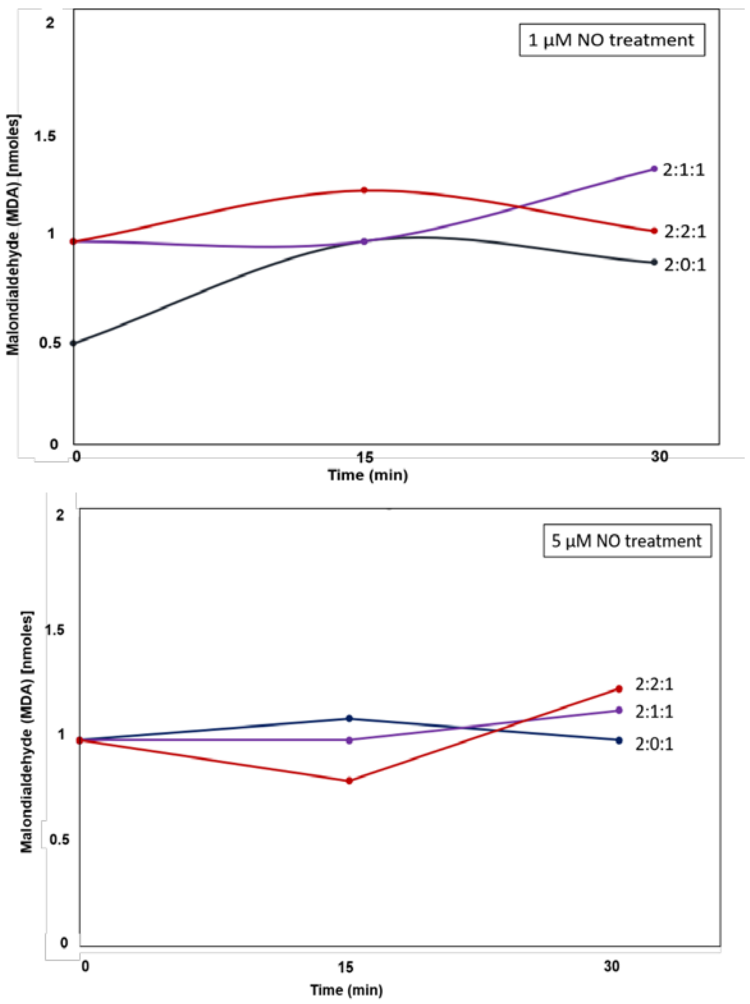

Figure S3: Lipid peroxidation assay to detect formation of MDA using N-palmitoyl-D-erythro-sphingosylphosphorylcholine (100% 16:0 SM) for different ratios. In Figure 6, we found that, increase in egg SM addition caused increase in MDA concentration. To confirm SM contribution in lipid peroxidation, egg SM was replaced with 100% 16:0 SM. The data presented here shows changes only due to POPC modifications the concentration of MDA detected without significant changes. Each data point is the average of three measurements.

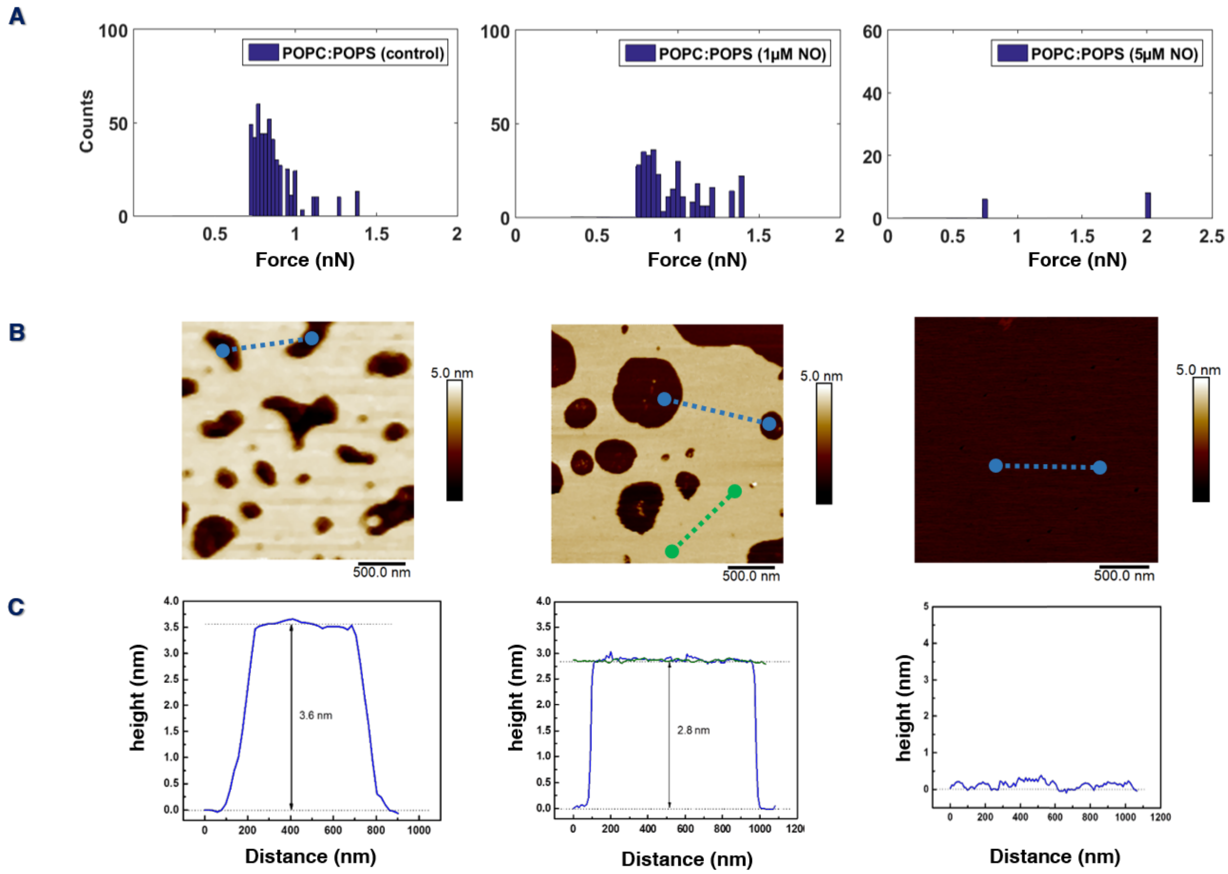

Figure S4: Pull-out force histogram (A), AFM images (B) and their corresponding height profiles (C) of POPC:POPS bilayer treated with 1 µM NO (*middle*) and 5 µM NO (*right*). POPC:POPS bilayer shows linear reduction in height of bilayer with increasing NO concentration. At 1 µM NO treatment, the maximum forces were similar to control (A, *left*), but complete membrane disruption was observed at 5 µM NO (A, *right*).

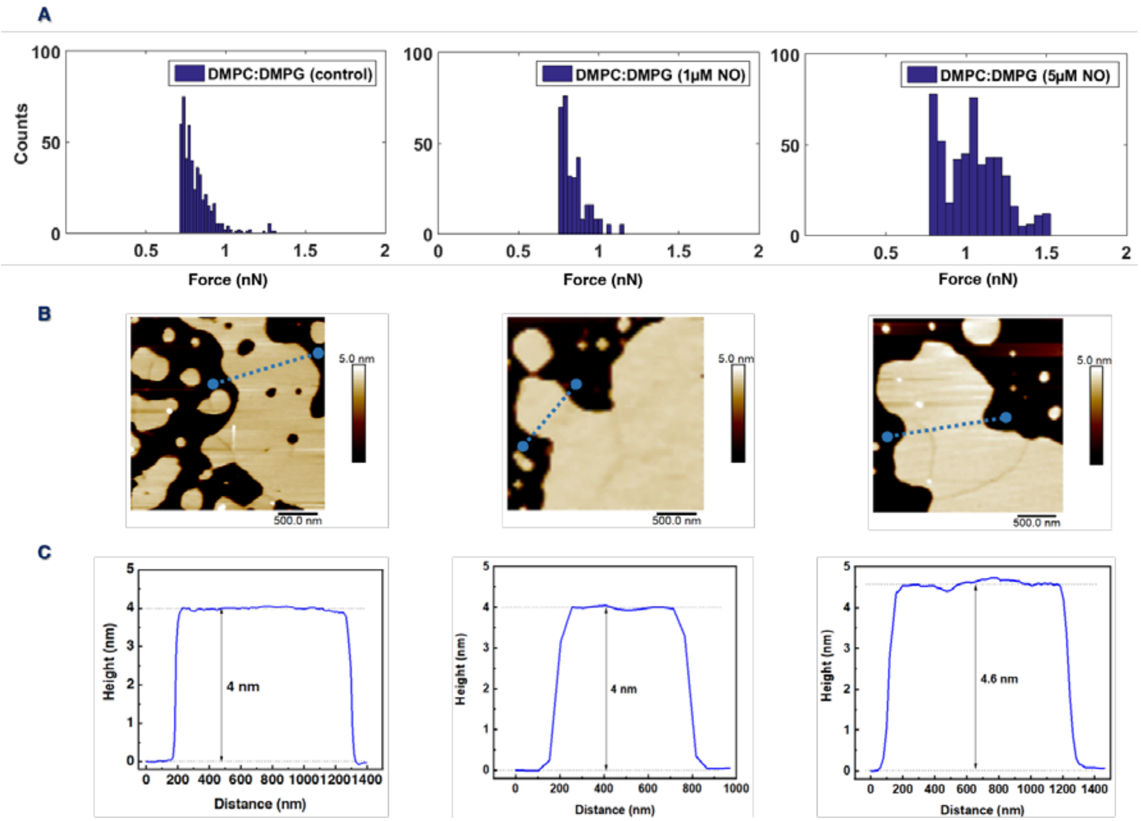

Figure S5: Pull-out force histogram (A), AFM images (B) and height profiles (C) of DMPC:DMPG bilayer treated with 1 µM NO (middle) and 5 µM NO (right). No changes in height or peak forces for 1 µM NO concentrations were found. At 5 µM NO, the maximum forces increased with increase in bilayer height, indicating NO dependent changes on saturated membranes.
