# Supplementary material for: Identification of a critical lipid ratio in raft-like phases exposed to nitric oxide: An AFM study
Source: Biophys J. 2021 Jun 29;120(15):3103–11. doi: 10.1016/j.bpj.2021.06.009 (PMC8390956; doi:10.1016/j.bpj.2021.06.009)
Supplement: Document S2. Article plus supporting material [file mmc2.pdf]

Biophysical Journal
Article

Biophysical Society

Identification of a critical lipid ratio in raft-like phases exposed to nitric oxide: An AFM study

Sanjai Karanth,[1,2] Amir Azinfar,[3] Christiane A. Helm,[3] and Mihaela Delcea[1,2,4,*]
[1]Institute of Biochemistry, University of Greifswald, Greifswald, Germany; [2]ZIK-HIKE, Zentrum für Innovationskompetenz "Humorale Immunreaktionen bei kardiovaskulären Erkrankungen", Greifswald, Germany; [3]Institute of Physics, University of Greifswald, Greifswald, Germany; and [4]DZHK (Deutsches Zentrum für Herz-Kreislauf-Forschung), partnersite Greifswald, Germany

ABSTRACT Lipid rafts are discrete, heterogeneous domains of phospholipids, sphingolipids, and sterols that are present in the cell membrane. They are responsible for conducting cell signaling and maintaining lipid-protein functionality. Redox-stress-induced modifications to any of their components can severely alter the mechanics and dynamics of the membrane causing impairment to the lipid-protein functionality. Here, we report on the effect of sphingomyelin (SM) in controlling membrane permeability and its role as a regulatory lipid in the presence of nitric oxide (NO). Force spectroscopy and atomic force microscopy imaging of raft-like phases (referring here to the coexistence of "liquid-ordered" and "liquid-disordered" phases in model bilayer membranes) prepared from lipids: 1-palmitoyl-2-oleoyl-glycero-3-phosphocholine (POPC):SM:cholesterol (CH) (at three ratios) showed that the adhesion forces to pull the tip out of the membrane increased with increasing SM concentration, indicating decreased membrane permeability. However, in the presence of NO radical (1 and 5 μM), the adhesion forces decreased depending on SM concentration. The membrane was found to be stable at the ratio POPC:SM:CH (2:1:1) even when exposed to 1 μM NO. We believe that this is a critical ratio needed by the raft-like phases to maintain homeostasis under stress conditions. The stability could be due to an interplay existing between SM and CH. However, at 5 μM NO, membrane deteriorations were detected. For POPC:SM:CH (2:2:1) ratio, NO displayed a pro-oxidant behavior and damaged the membrane at both radical concentrations. These changes were reflected by the differences in the height profiles of the raft-like phases observed by atomic force microscopy imaging. Malondialdehyde (a peroxidation product) detection suggests that lipids may have undergone lipid nitroxidation. The changes were instantaneous and independent of radical concentration and incubation time. Our study underlines the need for identifying appropriate ratios in the lipid rafts of the cell membranes to withstand redox imbalances caused by radicals such as NO.

SIGNIFICANCE Lipid rafts are specialized domains in the cell membrane and are critical for many cell transduction mechanisms. Any modifications to lipid rafts can alter their functionality, especially in stress conditions. Although studies on the effect of cholesterol concentration and its oxidation on membrane characteristics have been known, understanding the influence of sphingomyelin is equally important. Here, we look into whether nitric oxide radical can perturb the membrane permeability and bilayer thickness with changing sphingomyelin and nitric oxide concentration.

INTRODUCTION

Cell membrane can be understood as a specialized dynamic bilayer primarily composed of phospholipids, which can undergo various physical modulations in response to a biological process. The induced physical changes, for example, due to protein function (1,2) are reflected by changes in the mechanical properties of the cell membrane. Along with phospholipids, proteins and carbohydrates are other important components of the cell membrane (3) and form a frontline barrier in maintaining cell integrity. However, in a redox environment, phospholipids are highly susceptible and can undergo modifications (from a localized change (4) to complete membrane disruption). Generally, an exogenous or endogenous free radical attack on the phospholipids causes lipid peroxidation, a process that consists of three steps: 1) initiation, which includes formation of lipid peroxyl radicals (5); 2) propagation of peroxyl radicals; and 3) its termination. Each of these steps can cause significant changes to the chemical structure of phospholipids, wherein membrane functionality can either be impaired or lost. The propensity with which the lipids are modified

Submitted October 27, 2020, and accepted for publication June 2, 2021.
*Correspondence: delceam@uni-greifswald.de
Editor: Sarah Veatch.
https://doi.org/10.1016/j.bpj.2021.06.009
© 2021 Biophysical Society.
This is an open access article under the CC BY license (http://creativecommons.org/licenses/by/4.0/).
Check for updates

depends highly on the type of radical used. Such modifications of the phospholipids can be elucidated by detecting changes in the membrane forces (rupture or adhesion) as a parameter, among many others available (6–8). Such quantifications can help to understand properties such as membrane permeability (not to be confused with passive diffusion of molecules across a lipid bilayer) and instability (which can be measured with tip-membrane pull-out force), possible lateral movement of phospholipids, etc. An increase in the pull-out force indicates an increase in membrane rigidity and a decrease in membrane permeability.

Nitric oxide (NO) is one of the most intriguing radicals because of its ability to promote and inhibit lipid peroxidation (9). Although NO is not a strong oxidant and has a very short half-life, its action cannot be undermined especially under aqueous conditions. Its lipophilic nature makes it an interesting molecule for its action on lipid bilayers. Of special interest is its effect on lipid rafts, specialized microdomains in a cell membrane with distinct composition. They consist of a unique combination of phospholipids, sterols (e.g., cholesterol (CH)), and sphingolipids (10,11) and has been found to locally change the physical properties of a cell membrane. Unlike general phospholipid membranes, which are known to be "liquid-disordered" in their arrangement above transition temperature, presence of CH makes the membrane "liquid-ordered" (12–15). This phase change effectively reduces the lateral diffusion of biomolecules (e.g., proteins) in the bilayer, suggesting its crucial role (e.g., in fibrin clot retraction by integrin protein αiibβ3-mediated platelet aggregation (16) or as therapeutic targets (17)). As coexistence of "liquid-ordered" and "liquid-disordered" phases are defined in model bilayer membranes (18–21), we will refer to these phases as "raft-like phases" (R_LPs).

With many studies focused on understanding the effect of CH concentration on membrane properties under redox conditions (22–25), equal focus on interpreting the role of sphingomyelin (SM) is needed. With existing literature emphasizing on relation between sphingolipid metabolism and redox stress (26–31), little is understood on the final role of SM from a membrane organizational and physical perspective. Synthetic lipid systems (32) offer an alternative in detecting the influence of such different phases in the bilayer for elucidating their larger role in the cell.

Here, we combine force spectroscopy and atomic force microscopy (AFM) imaging to investigate the action of NO on R_LPs using POPC:SM:CH as model lipid composition. Although the phospholipid composition varies with each cell type, the outer leaflet of any cell membrane in eukaryotes mainly consists of phosphatidylcholine (33,34). For this reason, we have selected for this study the unsaturated phosphatidylcholine POPC lipid, which mimics the mammalian cell composition (32). We have characterized the membrane properties at varying SM concentrations and NO addition. In addition, a biochemical assay that detects malondialdehyde (MDA), an important stress biomarker, has been used.

MATERIALS AND METHODS

Formation of small unilamellar vesicles

Small unilamellar vesicles (SUVs) were prepared using phospholipids 1-palmitoyl-2-oleoyl-glycero-3-phosphocholine (POPC), egg SM, and natural CH (Avanti Polar Lipids, Alabaster, AL). Lipids were first solubilized in a solution containing chloroform that was mixed and dried under a stream of nitrogen and kept under vacuum overnight. Then, the dried lipid film was resuspended in PBS buffer (Biowest, Nuaillé, France) at pH 7.4 containing 1 mM $CaCl_2$ (Sigma-Aldrich, Taufkirchen, Germany). The lipid solution was then sonicated (SoniPrep 150 Plus; MSE Centrifuges, Nuaille, France) with a probe tip sonicator until the color of the solution turned from milky to clear ($\sim$5 min for the change in color). This solution was later centrifuged at 13,000 $\times$ g for 15 min to remove any titanium particles from the probe and the supernatant was collected. The lipid composition in the SUVs were varied: i.e., POPC:SM:CH in ratios of 2:0:1 (0.75 mM:0 mM:0.375 mM), 2:1:1 (0.75 mM:0.375 mM:0.375 mM), and 2:2:1 (0.75 mM:0.75 mM:0.375 mM). 100 μL of the preformed SUVs from each ratio were then diluted into 100 μL of PBS buffer. To prepare R_LPs from the above solution, 70 μL was taken and incubated onto freshly cleaved mica (area of 0.5 cm^2) sheet for 40 min at room temperature (RT) (which is above the lipid transition temperature) to form bilayers. The excess solution containing unbound vesicles were removed and fresh PBS buffer was added onto mica sheet for further experimentation.

NO action on lipid bilayers

The effect of NO on the bilayers was observed using the NO donor molecule: 1-hydroxy-2-oxo-3-(3-aminopropyl)-3-isopropyl-1-triazene called as NOC-5 (Dojindo, München, Germany). NOC-5 was added to the preformed lipid bilayers and allowed to interact for 15 min at RT. The time period was selected based on our previous findings (35) in which this time period was sufficient to observe a detectable physical change. Postincubation, the NOC-5 solution was aspirated, and fresh PBS buffer was added. The NO-treated bilayers were later subjected to AFM imaging and force spectroscopy. NOC-5 is a molecule which instantly releases NO when it comes in contact with H^+ ions in solution. Hence, it was prepared in 10 mM NaOH to reduce this instant release. However, as OH^- ions can impact the pH of the microenvironment, as per company suggestions, NOC-5 was added such that volume ratio does not exceed 1/50 of the total sample volume. This retained the overall pH of sample in solution. NOC-5 solution was freshly prepared for every measurement.

Force spectroscopy

Force spectroscopy measurements were carried in aqueous solution at RT to determine the forces of lipid bilayers in PBS buffer using JPK NanoWizard 3 (JPK Instruments AG, Berlin, Germany). Cantilevers (OBL-10, nominal spring constant of $\sim$6 pN nm^{-1} and nominal tip radius of 30 nm from Bruker, Ettlingen, Germany) were UV-ozone treated (Pro Cleaner Plus; BioForce Nanoscience, Virginia Beach, VA) for 30 min and calibrated using thermal method. The calibration was first done in air against mica and then in PBS buffer. The calibration software provided in the JPK instrument recorded the new spring constant and deflection sensitivity that was used further. A deviation of 10–20% from the company mentioned spring constant was observed. The force curves obtained (approach speed of 1 μm/s) were processed using JPK Data Processing software (version 5.0.91) and analyzed using a home-written MATLAB (The MathWorks, Natick, MA) script. To determine the peak adhesive forces, a histogram

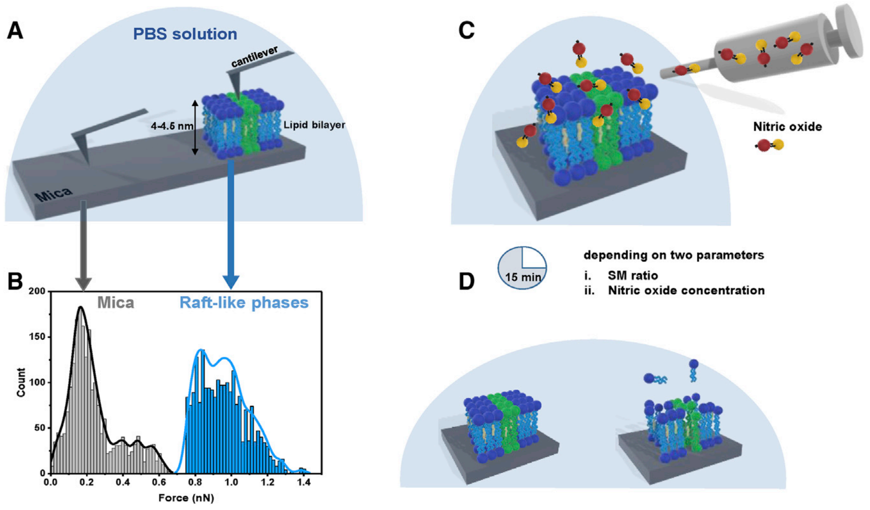

FIGURE 1 Overview of the experimental setup and determination of threshold pull-out forces. Before measurements of tip-membrane forces, forces due to nonspecific interactions (i.e., tip-mica surface) were determined (*A*). The tip-mica surface generated adhesive forces ranging between 0.02 and 0.65 nN (*B*) with a maximum at ~0.2 nN. Based on the obtained values, 0.75 nN was set as threshold force above which adhesive forces were attributed to the pull-out force of tip from the lipid bilayer. Control and R_LPs samples (with changing SM concentration) were later treated with NO for 15 min (*C*) and the possible outcomes were determined using force spectroscopy and AFM imaging (*D*). The histogram shown in (*B*) is plotted from the adhesion data of untreated POPC:SM:CH (2:0:1) bilayers. The straight lines are KDE. Break-through forces are described in Fig. 2. To see this figure in color, go online.

of the forces obtained was plotted and Kernel density estimation (KDE) method (nonparametric method for multivariate distribution analysis) was applied (with Gaussian kernel) to determine the peak position.

AFM imaging and data analysis

AFM imaging under tapping mode in liquid was carried out in Bioscope Resolve machine (Bruker) using FESP-V2 cantilevers (Bruker) with a nominal tip radius of 8–10 nm. Images of control and NO-treated samples were captured. The height profile of each of the samples were determined and analyzed using Nanoscope Analysis software v2.0 (Bruker). During analysis, only flatly adsorbed bilayers were considered. The roughness of both the substrate, i.e., mica, was determined.

MDA assay

MDA is an organic compound formed when phospholipids undergo lipid peroxidation. In this colorimetric assay (Biorbyt, Eching, Germany), SUVs were treated initially with NO for 15 min at RT, which were later allowed to react with thiobarbituric acid (TBA) molecule, which forms MDA-TBA adduct (indication of oxidation-based products). Absorbance of the formed adducts is determined at 532 nm. The quantification of MDA (nM) molecules was carried out as described in the kit's manual.

RESULTS AND DISCUSSION

Changes in the membrane permeability (referred as tip-membrane permeability in future text) by varying SM concentration and respective action by NO were investigated by measuring the adhesive forces (referred as pull-out force) and break-through forces. Fig. 1 shows an overview of the experimental setup.

A schematic representation of a force curve obtained from a force spectroscopy experiment is displayed in Fig. 2. As the AFM tip approaches and detects the lipid bilayer, an initial repulsive force is followed by a kink in the approach curve (Fig. 2, point "1") indicating the penetration of the AFM tip into the bilayer (36). When the tip is pulled out from the bilayer (retract curve), an adhesive force (i.e., pull-out force) is observed. The width (D) obtained in each force curve represents the thickness of the bilayer. The compressive force required to obtain the width indicates the break-through force. In our experiments, we measure pull-out and break-through forces. Before the start of experiments, baseline forces had to be determined to avoid nonspecific interactions i.e., background noise attributed by the equipment and tip-mica pull-out force (due to incomplete coverage of lipid bilayer on the mica, Fig. 1 *A*). Fig. 1 *B* shows the obtained forces. A background noise of 0.02 nN was fixed and the tip-mica surface interactions yielded a distribution of ranging between 0.02 and 0.65 nN with maximum at $\sim$0.2 nN (Fig. 1 *B*, *gray histogram*; Fig. S1). Based on the obtained values, an upper limit of 0.75 nN was set as threshold, above which measured forces were attributed to pull-out forces of the tip from the lipid bilayer. From the total force curves recorded, events for tip-bilayer interactions constituted less than 5%.

SM influences pull-out forces in R_LP

We first analyzed how SM impacts the pull-out forces after the tip penetration, which entails on tip-membrane permeability. Three different ratios were used for R_LP (i.e., POPC:SM:CH in ratios of 2:0:1, 2:1:1, and 2:2:1). For R_LP, we report two maxima of the pull-out forces with 2:0:1 ratio (i.e., in the absence of SM) at 0.83 and 1.0 nN with forces ranging between 0.75 and 1.38 nN (Fig. 3 *A*, *left*). The maxima of the forces were determined using KDE analysis. The data points that determined the maxima of the forces are those that had a probability density greater than 0.2 during analysis (representing significant events). With addition of SM, i.e., at 2:1:1 ratio, the pull-out force range was found to be similar to that of 2:0:1 ratio with two distinct maxima with one at 0.81 nN and other at 1.02

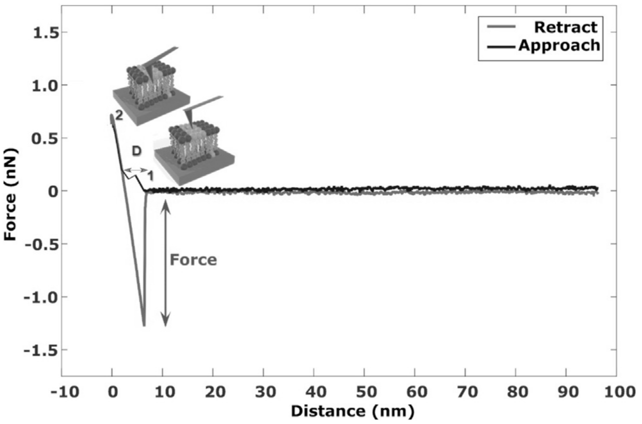

FIGURE 2 Schematic representation of acquisition of a force curve and respective events on the bilayer. Initially, the tip starts moving in the solution from a large distance toward the lipid bilayer (approach curve) until it comes in contact with it (indicated in the diagram as point 1). Then, the tip penetrates into the bilayer (generating break-through force) and constant compliance occurs (point 2). When the tip is pulled out of the lipid bilayer, it generates an adhesive force, detected as pull-out force (retract curve). The depth of tip penetration on approach (D), corresponds to the height of bilayer. As a typical example, a force curve for untreated POPC:SM:CH (2:0:1) bilayers is shown.

nN (Fig. 3 *B*, *left*). Upon increasing SM further, i.e., at 2:2:1 ratio, there was an increase in the maximum of the pull-out force to 2.04 nN (Fig. 3 *C*, *left*). Another important observation was that, unlike previous ratios in which the pull-out force spectrum was broad, most of the forces for 2:2:1 ratio was localized at around 2 nN. The increase in the pull-out forces with increased SM concentration indicates an increase in membrane rigidity and reduction in the tip-membrane permeability. When pull-out forces were compared with break-through forces (Fig. S2), 2:0:1 ratio showed a wide distribution with peak break-through force at 68 pN. With increase in SM, break-through force started to increase (i.e., 80 pN for 2:1:1 ratio and two peaks for 2:2:1 ratio, one at 80 pN and other 125 pN). The increase in break-through forces correlated with pull-out forces.

NO modulates SM-dependent tip-membrane pull-out force

After describing the effect of inclusion of SM into bilayers, we now look into the action of NO on R_LP. 1 and 5 μM concentrations of NO were used because they are found in cells (37,38) at any given time and direct oxidation by NO is observed at lower concentrations. On treatment of R_LP 2:0:1 (POPC:SM:CH) with 1 μM NO, we found that, with respect to control (in the absence of NO, Fig. 3 *A*, *left*), the force distribution became very broad, and the forces ranged between 0.75 and 2.5 nN and maxima at 0.85 and 1.21 nN (a maximum with reduced intensity) were obtained (Fig. 3 *A*, *middle*). An increase in pull-out forces was visible with increase in NO concentration to 5 μM with the force histogram ranged between 1.6 and 2.1 nN (Fig. 3 *A*, *right*) having peaks at 1.75 and 2.05 nN. These numbers collectively indicate that in case of R_LP without SM, addition of NO caused reduction in tip-membrane permeability.

ptUpon addition of SM to the R_LP i.e., in 2:1:1 (POPC:SM:CH) ratio, a competitive behavior was observed compared to 2:0:1 (POPC:SM:CH) ratio. When the bilayers were treated with 1 μM NO, the force range was similar to that of sample in absence of NO (Fig. 3 *B*, *left*) and the peak maxima were at 0.82, 0.95, and 1.12 nN (Fig. 3 *B*, *middle*). However, treatment of the bilayer with 5 μM NO showed only one maximum at 0.89 nN with the forces ranging between 0.75 and 1.6 nN (Fig. 3 *B*, *right*). Although pull-out forces between 1 and 1.5 nN were captured for 2:1:1 (POPC:SM:CH) ratio, the number of events was too low for quantification. No significant shifts in the peaks or the force range were visible. At 2:2:1 (POPC:SM:CH) ratio, 1 μM NO treatment caused sufficient membrane damage i.e., increased tip-membrane permeability (opposite to previous ratios). The maximal force was obtained at 0.80 nN (Fig. 3 *C*, *middle*), whereas control (Fig. 3 *C*, *left*) showed pull-out forces at 2 nN. The increase in tip-membrane permeability continued for treatment with 5 μM NO as well (Fig. 3 *C*, *right*) with peak at 0.78 nN (similar to 1 μM NO). These results confirm that as the concentration of SM is increased, membrane stability is significantly perturbed by NO radical. Break-through forces of NO action on R_LP (Fig. S2) also showed a similar behavior. For 2:0:1 ratio, peak break-through force was found at 68 pN for 1 μM NO and 50 pN for 5 μM NO. At 2:1:1, slight reduction in the peak break-through force from 80 pN (control) to 75 pN at 1 μM NO and at 5 μM NO two peaks, one at 48 and 80 pN was detected. It was observed that a constant break-through force at 80 pN was obtained throughout. However, this was not the case with 2:2:1 as it showed significant reduction in the break-through forces to 27 pN at 1 μM NO and 32.5 pN for 5 μM NO compared with control. These changes highlight that, NO along with SM starts to show a concentration dependence and start behaving as a regulatory molecule. To validate these observations further, AFM imaging of the R_LP was performed to observe any possible changes to topology.

AFM imaging of R_LPs

Fig. 4 shows AFM images and height histograms for different lipid ratios (before and after NO treatment). At 2:0:1 (POPC:SM:CH) ratio, AFM imaging showed presence of a single height peak (4.1 nm) indicating an homogeneous phase (Fig. 4 *A*, *left*). The bilayer height upon treatment with 1 μM NO was 1.76 nm (Fig. 4 *A*, *middle*). This behavior continued when NO concentration was increased to 5 μM with the height being reduced further to 1.61 nm (Fig. 4 *A*, *right*). The decrease in the height profiles for 2:0:1 (POPC:SM:CH) ratio (showing membrane fluctuations) were in sharp contrast with the force data (in which pull-out forces increased with NO addition).

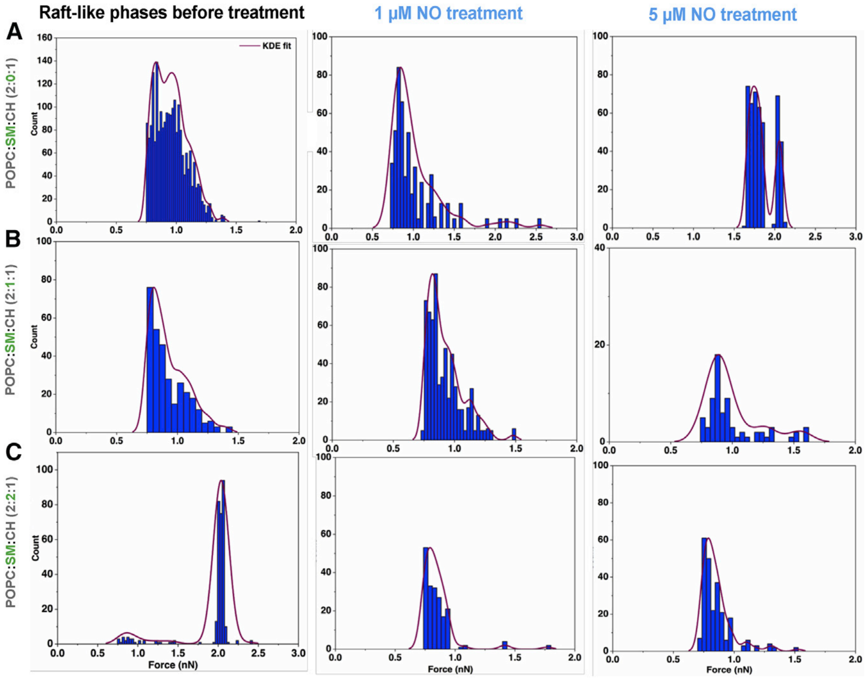

FIGURE 3 Histograms of tip-lipid bilayer pull-out forces of R_LPs with different SM concentration in dependence to NO treatment. Force data of untreated R_LP (*left column*), R_LP post 1 μM (*middle column*) and post 5 μM NO treatment (*right column*) are shown. In each row, R_LP with the same composition are shown. Untreated R_LP show increase in pull-out forces at the largest SM concentration (*A–C*, *left*) which indicates reduced membrane permeability. Addition of 1 μM NO to 2:0:1 (POPC:SM:CH) ratio caused the force spectrum to broaden (*A*, *middle*) compared with control (*A*, *left*), with maxima forces at 0.85 and 1.21 nN. At 5 μM, the maxima forces increased further to 1.74 and 2.05 nN (*A*, *right*). For 2:1:1 (POPC:SM:CH) ratio, a competitive behavior is observed and after NO treatment (at 1 and 5 μM), the pull-out forces (*B*, *middle* and *right*) were similar to untreated 2:1:1 (POPC:SM:CH) ratio. This indicates presence of possible critical concentration in maintaining membrane integrity. At 2:2:1 (POPC:SM:CH) ratio, detrimental effect of NO is observed (*C*, *middle* and *right*) with decrease in pull-out forces compared with control (*C*, *left*), indicating membrane destruction. Histograms represent force data of only tip-membrane interactions and tip-mica forces are excluded. To see this figure in color, go online.

With addition of SM, the height distribution analysis of AFM images (Fig. 4 *B*) showed two peaks as expected (32), indicating the coexistence of two phases in R_LP. The difference in heights between these two phases was around 0.5 nm (Fig. 4, *B* and *C*, *left*). At 2:1:1 (POPC:SM:CH) ratio, we found that with NO treatment, the height difference between the lipid phases were minor (Fig. 4 *B*, *middle* and *right*) and similar to that of control. However, the thickness of each phase was reduced i.e., height of one lipid phase decreased from 4.46 nm (control) to 4.30 nm (1 μM NO) and 3.76 nm (5 μM NO) and the other from 3.93 nm (control) to 3.88 nm (1 μM NO) and 3.25 nm (5 μM NO). The height information of 2:1:1 ratio was in accordance with the forces (pull-out and break-through) confirming maintenance of membrane integrity (some sort of equilibrium) for this particular R_LP composition.

For 2:2:1 (POPC:SM:CH) ratio, firstly, both phases are thinner than the 2:1:1 ratio (3.97 and 3.36 nm compared with 4.46 and 3.93 nm, respectively, Fig. 4 *C left*). Treatment with NO showed disappearance of two distinct phases in the bilayer (Fig. 4 *C*, *middle* and *right*) with the height reduced to 3 nm (for 1 μM NO, Fig. 4 *C*, *middle*) and 2.75 nm (for 5 μM NO, Fig. 4 *C*, *right*). Merging of two phases at 2:2:1 ratio indicates definite structural reorganization in the R_LPs. Similar observations were made in the force data as well (i.e., decrease in the pull-out and break-through forces). This difference, when compared to 2:1:1 (POPC:SM:CH) ratio, underlines the influence of lipid composition on stability to NO exposure. It also indicates that action of NO becomes regulatory in presence of SM and is moving toward becoming increasingly pro-oxidant with increase in SM concentration.

Fig. 5 summarizes the behavior of tip-membrane permeability for all three different R_LP ratios at investigated NO concentrations. In the presence of NO (*blue arrow*), tip-membrane permeability of POPC:SM:CH (2:0:1) starts to decrease with addition of NO (i.e., the pull-out force increases). POPC:SM:CH (2:1:1) ratio showed mixed results i.e., at 1 μM NO, the R_LP were stable and tip-membrane permeability was unaltered but at 5 μM NO, the raft-like phase became slightly unstable however, the tip-membrane permeability was almost the same. This suggests the existence of a possible critical ratio (*violet-dashed box*) in natural cell membranes in response to change in redox conditions. The critical ratio represents the most stable composition. POPC:SM:CH (2:2:1) ratio showed increase in tip-membrane permeability with increase in NO concentration.

Lipid composition dependent physicochemical modifications

A phospholipid undergoes modification when a radical attack either the headgroup or tail group or both. We believe that, here, the changes are restricted to the phospholipid tail. The reason is that NO by itself is a lipophilic molecule and has greater partition coefficient into the hydrophobic spaces of the membrane when compared with other solute molecules of similar size. This understanding is supported in literature both by experiments and simulations

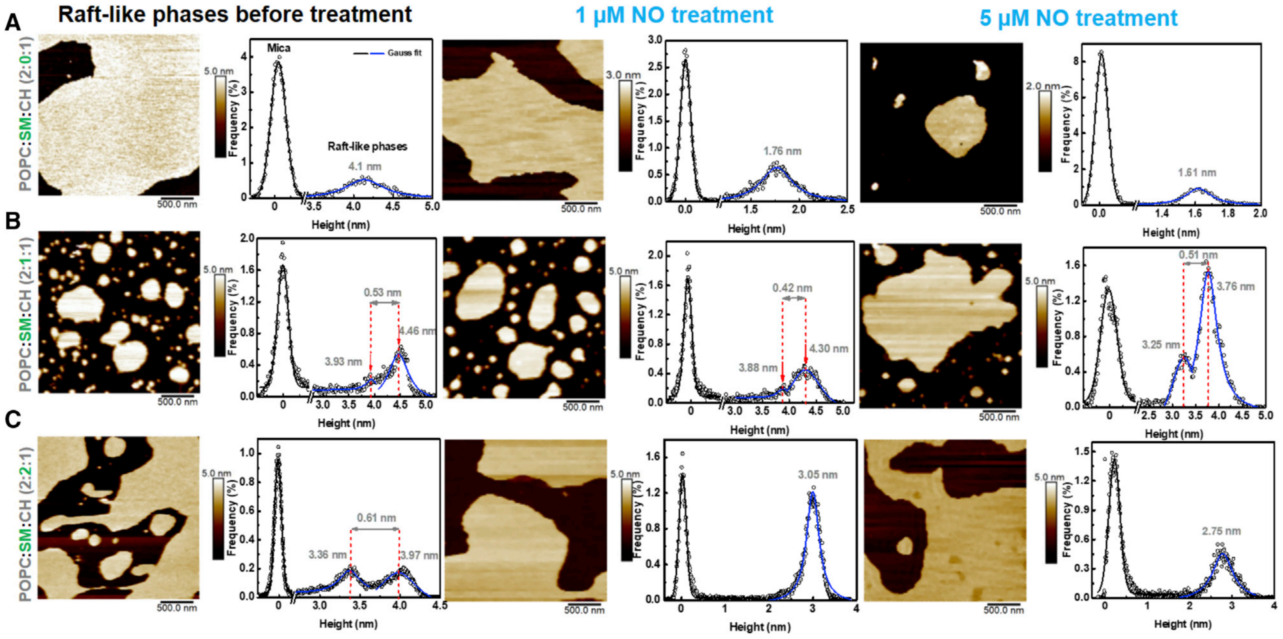

FIGURE 4 AFM images of R_LP with changing SM concentration and NO treatment. Images of untreated R_LP (*left column*), R_LP post 1 μM (*middle column*) and post 5 μM NO treatment (*right column*) are shown. The height histograms of each image are adjacent to it. At 2:0:1 (POPC:SM:CH) ratio, addition of NO showed decrease in the height of the lipid bilayer (*A*, *middle* and *right*) compared with control (*A*, *left*). At 2:1:1 (POPC:SM:CH) ratio, the difference in the heights of the two lipid phases for NO-treated samples was minor (*B*, *middle* and *right*) and similar to control (*B*, *left*) i.e., ~0.5 nm even though reduction in the height of individual phases were observed. This indicates membrane stability and negligible effect of NO. At 2:2:1 (POPC:SM:CH) ratio, merging of lipid phases (*C*, *middle* and *right*) is observed indicating the role of NO as a pro-oxidant. To see this figure in color, go online.

(22,39,40). With the presence of C=C bond in the phospholipid tail region, lipid peroxidation by NO is driven mainly by either nitration, nitroxidation, or both (41–43) unlike other radicals where one kind of chemical reaction takes place (e.g., hydroxyl that causes only oxidative reaction and attacks mainly the headgroup of phospholipid).

Although we cannot confirm on the exact type of chemical modifications undergone by the R_LP, we performed peroxidation assay to detect MDA in the system. MDA is a product formed because of lipid modification mainly through oxidation and is a standard biomarker used for detection of oxidative stress in cells. We found that in R_LP with 2:0:1 (POPC:SM:CH) ratio, ~0.6 nM of MDA for 1 μM NO and ~0.4 nM of MDA for 5 μM NO was formed. This amount increased to ~0.8 and ~0.75 nM (1 and 5 μM NO) for 2:1:1 (POPC:SM:CH) ratio and ~1.25 and ~1.4 nM (1 and 5 μM NO) for 2:2:1 (POPC:SM:CH) ratio (Fig. 6).

This increase in MDA clearly indicates that lipids underwent nitroxidation (i.e., oxidation by NO). Interestingly, the increase in MDA is also attributed to the unsaturation present in the SM. Because we used egg SM (which is 95% saturated, 3% unsaturated, and 2% unknown), increase in MDA with increase in SM concentration was detected. To confirm this effect, MDA detection was carried out with N-palmitoyl-D-erythro-sphingosylphosphorylcholine (100% saturated) suggested that influence of SM was negligible, and oxidation could be mainly attributed to POPC molecules (Fig. S3). By comparing the amount of MDA formed (Fig. 6, *above* and *below*) for 1 and 5 μM NO treatment, we found that the difference between the obtained values are not significantly varied indicating that the MDA formation is independent of NO concentration or radical exposure time. Two possibilities arise here: 1) all phospholipid molecules underwent initial chemical changes, and 2) NO radical itself underwent modifications (as it is not a strong oxidant and no further increase in MDA was observed at 30 min).

Based on these results, we interpret that changes in the membrane mechanical properties of R_LP highly vary. This behavior was absent when compared with simple unsaturated phospholipid bilayers (e.g., POPC/POPS in which radical attack showed a linear change in height and decrease in pull-out forces, Fig. S4) or saturated bilayers (e.g., DMPC/DMPG in which 1 μM NO stabilized the membrane and 5 μM NO increased tip-membrane permeability, Fig. S5). This was evident with other similar phospholipids as well (22,44). In terms of lipid packing between unsaturated phospholipids (POPC) and sphingolipids (SM), the tail region of sphingolipids is elongated and contacts adjacent sphingolipid molecules thereby, increasing the van der Waal attraction forces between the tails (45–47). Such increased forces reduced the tip-membrane permeability as observed in bilayers with large SM concentration (Fig. 3). As R_LP also contains sterol (CH); this mixture underlines the possible interplay by SM and CH in the presence of NO. When looked individually, CH is known to

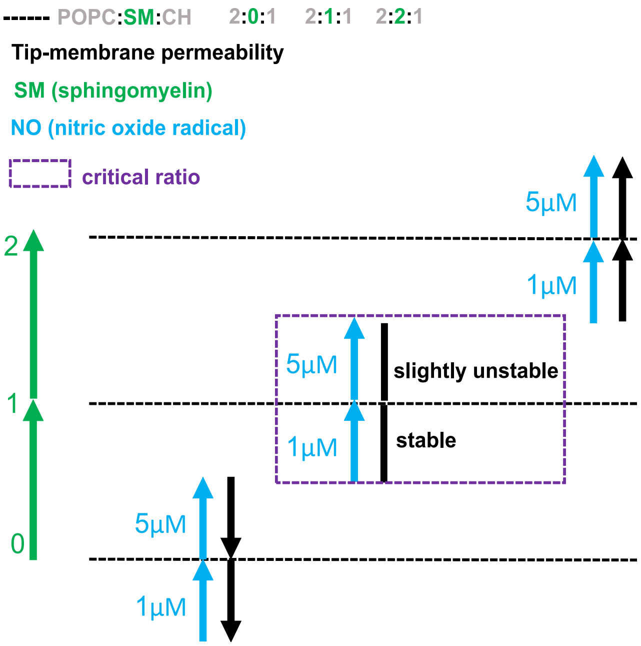

FIGURE 5 Interpretation of tip-membrane permeability of R_LPs with changing SM ratio and NO concentration. In the presence of NO (*blue arrow*), tip-membrane permeability (*black arrow*) of POPC:SM:CH (2:0:1) decreases with increase in NO concentration. With addition of SM (*green arrow*) i.e., POPC:SM:CH (2:1:1) ratio, 1 μM NO shows stable R_LPs and unaltered tip-membrane permeability. At 5 μM NO, the tip-membrane permeability was almost the same. This indicates existence of a critical ratio (*violet-dashed box*). POPC:SM:CH (2:2:1) ratio showed increase in tip-membrane permeability with increase in NO concentration. To see this figure in color, go online.

increase the thickness of lipid bilayer (48) by stretching its tails, but maintaining the chain volume (49) and causing close packing of lipid molecules (i.e., decreased molecular area). However, this does not occur in R_LP of 2:0:1 (POPC:SM:CH) ratio in presence of NO because we observe a decrease in the bilayer thickness, but an increase in pull-out forces. If CH had undergone nitroxidation, then it would be modified to oxysterols. There are many end products formed when CH is chemically modified into oxysterols and are primarily grouped as either tail-oxidized sterols or ring-oxidized sterols. Free radicals are known to cause mainly ring-oxidized sterols and these modified CH molecules do not significantly change the membrane permeability (50,51). However, possible change in the spatial orientations of these oxidized sterols cannot be overlooked (which could explain the reduction in the bilayer height postmodification to POPC). When SM was introduced i.e., in 2:1:1 (POPC:SM:CH) ratio, we reported that the membrane integrity was maintained at 1 μM NO and 5 μM NO exposure. In cell systems, there is evidence that SM sustains the redox homeostasis (52), but explicit information that a critical SM concentration is needed for sustainability, over which it starts to have deleterious effect is reported here for the first time. Available literature mentions that based on the structure of sphingolipids, they can participate in both inter- and intramolecular hydrogen bonding, which is not possible in glycerolipids. This assists in maintaining the membrane stability under stress (53); however, it depends on the extent of oxidation undergone by the SM (54). This was clearly visible when the membrane characteristics of R_LP 2:1:1 (POPC:SM:CH) ratio and 2:2:1 (POPC:SM:CH) ratio at two different NO concentrations were compared. Also, the presence of SM is known to inhibit oxidation of CH significantly (29), which explains increase in MDA formation (due to increasing exposure of lipid unsaturation by SM and POPC molecules), but not substantial variations in the heights of bilayer. This leads to our understanding that upon NO addition, SM along with POPC starts to dominate in the interplay with CH in the final outcome of membrane modifications rather than each of the molecules when observed individually.

Nitration of phospholipids depends on radical environment

Although we mentioned about nitroxidation previously (Fig. 6), phospholipid nitration (i.e., modification to phospholipid by reactive nitrogen moiety) is another possible modification that NO can induce on lipid bilayer. NO is a weak radical and usually not all the NO released by NOC-5 solution will cause direct membrane modification. It can easily be converted to other stable intermediates when reacted with other molecules depending on the surrounding environment (e.g., in presence of enzymes like glutathione peroxidase, radicals like hydroxyl or superoxide) (55,56). One such molecule which is present in our system is molecular oxygen. By itself, molecular oxygen is hydrophobic in nature and can reside in the intermediate spaces of membrane similar to NO. Because force measurements and imaging experiments were carried out in aqueous aerobic solution for 15 min, we assume that the time period was sufficient for NO to undergo reaction with oxygen. The stable products formed could be either nitrite (NO_2^-), nitrate (NO_3^-), or N_2O_3. Previously, we had reported on the formation of nitrite for the above used time duration (35). Hence, it is possible that these products can also constitute as potential nitrosating agents, which can cause nitro-fatty-acid generation (57–59). However, a detailed chemical analysis is required to determine the influence of these products on R_LPs.

CONCLUSIONS

In this study, combining biophysical and biochemical analyses, we show that NO radical can significantly alter the membrane characteristics and change the tip-membrane permeability and thickness of R_LPs. The membrane modifications were found to be dependent on the lipid composition and NO concentration. When the SM was varied between three ratios of POPC:SM:CH bilayers (i.e., 2:0:1, 2:1:1, and 2:2:1) we found that in the absence of SM, NO

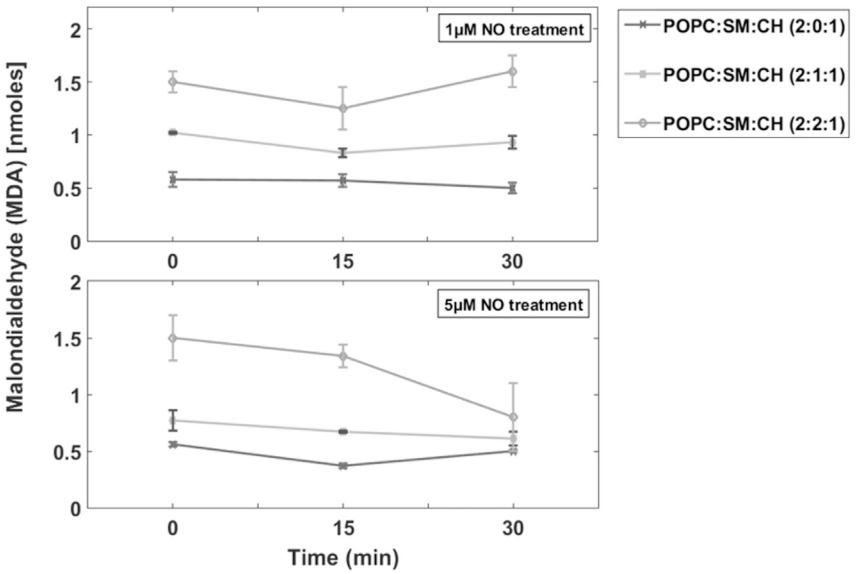

FIGURE 6 Lipid peroxidation assay to detect formation of MDA. With concentration of POPC being fixed, increasing SM supplemented to the amount of MDA (nM) formed. At 1 μM NO treatment (*above*), R_LP of 2:0:1 showed lower MDA concentration (~0.6 nM) compared to 2:1:1 (~0.8 nM) and 2:1:1 (POPC:SM:CH) showed lower MDA compared to 2:2:1 (POPC:SM:CH) ratio (~1.25 nM). The same behavior was observed at 5 μM NO concentration (*below*). The increase in MDA with increase in SM concentration may be due to the presence of unsaturation. To confirm this hypothesis, a similar assay was performed with N-palmitoyl-D-erythro-sphingosylphosphorylcholine (100% 16:0 SM) and constant MDA levels were observed irrespective of lipid ratio or NO concentration (Fig. S3). The assay shows that although NO initiated the peroxidation, the concentration of produced MDA becomes constant over time, indicating that all available unsaturated lipids were modified. The absorbance values were obtained after subtraction of the blank as mentioned in the Materials and methods.

increased the pull-out forces and decreased the tip-membrane permeability and membrane thickness. At 2:1:1 (POPC:SM:CH) ratio, membrane integrity is maintained (when compared with control) even with NO addition. However, the thickness of each phase in the R_LPs is reduced but the height difference between the phases was maintained (as observed at 5 μM NO). This indicates the presence and need for a critical ratio in the natural membranes to continue its functionality even under stress. An interplay between SM and CH is believed to be involved in maintaining the stability in which SM seems to dominate along with POPC. At 2:2:1 (POPC:SM:CH) ratio, the membrane thickness and stability were reduced with different phases being merged (i.e., single height) and the resultant phase being thinner than the control, indicating regulatory role of SM and NO. Peroxidation assay highlighted that lipids might have undergone nitroxidation and the extent of lipid modification due to peroxidation depended on SM concentration. In addition, the action of NO on the R_LPs was instantaneous and independent of incubation time and radical concentration.

SUPPORTING MATERIAL

Supporting material can be found online at https://doi.org/10.1016/j.bpj.2021.06.009.

AUTHOR CONTRIBUTIONS

S.K. and M.D. are responsible for design and conceptualization. S.K. performed force spectroscopy measurements, related data analysis, and preparation of figures. A.A. performed AFM imaging and analysis. S.K. and A.A. jointly analyzed and finalized imaging figures. S.K. wrote the manuscript. S.K., A.A., C.A.H., and M.D. are responsible for critical evaluation and reviewing the manuscript. S.K. and A.A. share the authorship for this manuscript.

ACKNOWLEDGMENTS

This work was funded by the Deutsche Forschungsgemeinschaft (DFG, German Research Foundation) Research Training Group (RTG) 1947 (231396381/GRK1947/3).

REFERENCES

1. Anishkin, A., S. H. Loukin, …, C. Kung. 2014. Feeling the hidden mechanical forces in lipid bilayer is an original sense. *Proc. Natl. Acad. Sci. USA*. 111:7898–7905.
2. Diz-Muñoz, A., D. A. Fletcher, and O. D. Weiner. 2013. Use the force: membrane tension as an organizer of cell shape and motility. *Trends Cell Biol.* 23:47–53.
3. van Meer, G., and A. I. de Kroon. 2011. Lipid map of the mammalian cell. *J. Cell Sci.* 124:5–8.
4. Karanth, S., B. Meesaragandla, and M. Delcea. 2020. Changing surface properties of artificial lipid membranes at the interface with biopolymer coated gold nanoparticles under normal and redox conditions. *Biophys. Chem.* 267:106465.
5. Ayala, A., M. F. Muñoz, and S. Argüelles. 2014. Lipid peroxidation: production, metabolism, and signaling mechanisms of malondialdehyde and 4-hydroxy-2-nonenal. *Oxid. Med. Cell. Longev.* 2014:360438.
6. Crawford, G. E., and J. C. Earnshaw. 1987. Viscoelastic relaxation of bilayer lipid membranes. Frequency-dependent tension and membrane viscosity. *Biophys. J.* 52:87–94.
7. Espinosa, G., I. López-Montero, …, D. Langevin. 2011. Shear rheology of lipid monolayers and insights on membrane fluidity. *Proc. Natl. Acad. Sci. USA*. 108:6008–6013.
8. Tyler, W. J. 2012. The mechanobiology of brain function. *Nat. Rev. Neurosci.* 13:867–878.
9. Hogg, N., and B. Kalyanaraman. 1999. Nitric oxide and lipid peroxidation. *Biochim. Biophys. Acta*. 1411:378–384.
10. Munro, S. 2003. Lipid rafts: elusive or illusive? *Cell*. 115:377–388.
11. Calder, P. C., and P. Yaqoob. 2007. Lipid rafts–composition, characterization, and controversies. *J. Nutr.* 137:545–547.
12. Xiang, T. X., and B. D. Anderson. 1998. Influence of chain ordering on the selectivity of dipalmitoylphosphatidylcholine bilayer membranes for permeant size and shape. *Biophys. J.* 75:2658–2671.
13. Thewalt, J. L., and M. Bloom. 1992. Phosphatidylcholine: cholesterol phase diagrams. *Biophys. J.* 63:1176–1181.

14. Owicki, J. C., and H. M. McConnell. 1980. Lateral diffusion in inhomogeneous membranes. Model membranes containing cholesterol. *Biophys. J.* 30:383–397.

15. Ohvo-Rekilä, H., B. Ramstedt, …, J. P. Slotte. 2002. Cholesterol interactions with phospholipids in membranes. *Prog. Lipid Res.* 41:66–97.

16. Bodin, S., C. Soulet, …, B. Payrastre. 2005. Integrin-dependent interaction of lipid rafts with the actin cytoskeleton in activated human platelets. *J. Cell Sci.* 118:759–769.

17. Rabani, V. 2017. Lipid rafts of platelet membrane as therapeutic target: role of OMICS. PhD thesis (Universite Bourgogne- Franche Comte).

18. Kahya, N., D. Scherfeld, …, P. Schwille. 2003. Probing lipid mobility of raft-exhibiting model membranes by fluorescence correlation spectroscopy. *J. Biol. Chem.* 278:28109–28115.

19. Samsonov, A. V., I. Mihalyov, and F. S. Cohen. 2001. Characterization of cholesterol-sphingomyelin domains and their dynamics in bilayer membranes. *Biophys. J.* 81:1486–1500.

20. Dietrich, C., L. A. Bagatolli, …, E. Gratton. 2001. Lipid rafts reconstituted in model membranes. *Biophys. J.* 80:1417–1428.

21. Bagatolli, L. A., and E. Gratton. 2000. Two photon fluorescence microscopy of coexisting lipid domains in giant unilamellar vesicles of binary phospholipid mixtures. *Biophys. J.* 78:290–305.

22. Yusupov, M., K. Wende, …, A. Bogaerts. 2017. Effect of head group and lipid tail oxidation in the cell membrane revealed through integrated simulations and experiments. *Sci. Rep.* 7:5761.

23. Roy, S., A. Dasgupta, …, O. Singh. 2016. Role of membrane cholesterol and lipid peroxidation in regulating the Na^+/K^+-ATPase activity in schizophrenia. *Indian J. Psychiatry.* 58:317–325.

24. Jacob, R. F., and R. P. Mason. 2005. Lipid peroxidation induces cholesterol domain formation in model membranes. *J. Biol. Chem.* 280:39380–39387.

25. Van der Paal, J., E. C. Neyts, …, A. Bogaerts. 2016. Effect of lipid peroxidation on membrane permeability of cancer and normal cells subjected to oxidative stress. *Chem. Sci. (Camb.).* 7:489–498.

26. Holthuis, J. C., T. Pomorski, …, G. Van Meer. 2001. The organizing potential of sphingolipids in intracellular membrane transport. *Physiol. Rev.* 81:1689–1723.

27. Ravandeh, M., G. Coliva, …, K. Wende. 2021. Protective role of sphingomyelin in eye lens cell membrane model against oxidative stress. *Biomolecules.* 11:276.

28. Oborina, E. M., and M. C. Yappert. 2003. Effect of sphingomyelin versus dipalmitoylphosphatidylcholine on the extent of lipid oxidation. *Chem. Phys. Lipids.* 123:223–232.

29. Sargis, R. M., and P. V. Subbaiah. 2006. Protection of membrane cholesterol by sphingomyelin against free radical-mediated oxidation. *Free Radic. Biol. Med.* 40:2092–2102.

30. Coliva, G., M. Lange, …, M. Fedorova. 2020. Sphingomyelins prevent propagation of lipid peroxidation—LC-MS/MS evaluation of inhibition mechanisms. *Molecules.* 25:1925.

31. Koval, M., and R. E. Pagano. 1991. Intracellular transport and metabolism of sphingomyelin. *Biochim. Biophys. Acta.* 1082:113–125.

32. Chiantia, S., N. Kahya, and P. Schwille. 2005. Dehydration damage of domain-exhibiting supported bilayers: an AFM study on the protective effects of disaccharides and other stabilizing substances. *Langmuir.* 21:6317–6323.

33. van Meer, G., D. R. Voelker, and G. W. Feigenson. 2008. Membrane lipids: where they are and how they behave. *Nat. Rev. Mol. Cell Biol.* 9:112–124.

34. Casares, D., P. V. Escribá, and C. A. Rosselló. 2019. Membrane lipid composition: effect on membrane and organelle structure, function and compartmentalization and therapeutic avenues. *Int. J. Mol. Sci.* 20:2167.

35. Karanth, S., and M. Delcea. 2020. Nitrosative stress affects the interaction of integrin alphaIIbbeta3 with its ligands. *Biochim. Biophys. Acta Biomembr.* 1862:183198.

36. Franz, V., S. Loi, …, H.-J. Butt. 2002. Tip penetration through lipid bilayers in atomic force microscopy. *Colloids Surf. B Biointerfaces.* 23:191–200.

37. Lancaster, J. R., Jr. 1997. A tutorial on the diffusibility and reactivity of free nitric oxide. *Nitric Oxide.* 1:18–30.

38. Malinski, T., Z. Taha, …, P. Tomboulian. 1993. Diffusion of nitric oxide in the aorta wall monitored in situ by porphyrinic microsensors. *Biochem. Biophys. Res. Commun.* 193:1076–1082.

39. Wennberg, C. L., D. van der Spoel, and J. S. Hub. 2012. Large influence of cholesterol on solute partitioning into lipid membranes. *J. Am. Chem. Soc.* 134:5351–5361.

40. Möller, M., H. Botti, …, A. Denicola. 2005. Direct measurement of nitric oxide and oxygen partitioning into liposomes and low density lipoprotein. *J. Biol. Chem.* 280:8850–8854.

41. Rubbo, H., R. Radi, …, B. A. Freeman. 1994. Nitric oxide regulation of superoxide and peroxynitrite-dependent lipid peroxidation. Formation of novel nitrogen-containing oxidized lipid derivatives. *J. Biol. Chem.* 269:26066–26075.

42. Reis, A., and C. M. Spickett. 2012. Chemistry of phospholipid oxidation. *Biochim. Biophys. Acta.* 1818:2374–2387.

43. O'Donnell, V. B., J. P. Eiserich, …, B. A. Freeman. 1999. Nitration of unsaturated fatty acids by nitric oxide-derived reactive nitrogen species peroxynitrite, nitrous acid, nitrogen dioxide, and nitronium ion. *Chem. Res. Toxicol.* 12:83–92.

44. Riske, K. A., T. P. Sudbrack, …, R. Itri. 2009. Giant vesicles under oxidative stress induced by a membrane-anchored photosensitizer. *Biophys. J.* 97:1362–1370.

45. McIntosh, T. J., S. A. Simon, …, C. H. Huang. 1992. Structure and cohesive properties of sphingomyelin/cholesterol bilayers. *Biochemistry.* 31:2012–2020.

46. Demel, R. A., and B. De Kruyff. 1976. The function of sterols in membranes. *Biochim. Biophys. Acta.* 457:109–132.

47. Brown, R. E. 1998. Sphingolipid organization in biomembranes: what physical studies of model membranes reveal. *J. Cell Sci.* 111:1–9.

48. Nezil, F. A., and M. Bloom. 1992. Combined influence of cholesterol and synthetic amphiphillic peptides upon bilayer thickness in model membranes. *Biophys. J.* 61:1176–1183.

49. Hung, W.-C., M.-T. Lee, …, H. W. Huang. 2007. The condensing effect of cholesterol in lipid bilayers. *Biophys. J.* 92:3960–3967.

50. Kulig, W. T., H. Mikkolainen, …, P. Jungwirth. 2017. Oxidation of cholesterol changes the permeability of lipid membranes. *Biophys. J.* 112:377A.

51. Kulig, W., A. Olżyńska, …, P. Jungwirth. 2015. Cholesterol under oxidative stress-How lipid membranes sense oxidation as cholesterol is being replaced by oxysterols. *Free Radic. Biol. Med.* 84:30–41.

52. Won, J.-S., and I. Singh. 2006. Sphingolipid signaling and redox regulation. *Free Radic. Biol. Med.* 40:1875–1888.

53. Pascher, I. 1976. Molecular arrangements in sphingolipids. Conformation and hydrogen bonding of ceramide and their implication on membrane stability and permeability. *Biochim. Biophys. Acta.* 455:433–451.

54. Borchman, D., O. P. Lamba, …, M. C. Yappert. 1992. The dual effect of oxidation on lipid bilayer structure. *Lipids.* 27:261–265.

55. Liu, X., M. J. Miller, …, J. R. Lancaster, Jr. 1998. Accelerated reaction of nitric oxide with O2 within the hydrophobic interior of biological membranes. *Proc. Natl. Acad. Sci. USA.* 95:2175–2179.

56. Violi, F., R. Marino, …, L. Loffredo. 1999. Nitric oxide and its role in lipid peroxidation. *Diabetes Metab. Res. Rev.* 15:283–288.

57. Bakan, E., S. Taysi, …, M. Gumus. 2002. Nitric oxide levels and lipid peroxidation in plasma of patients with gastric cancer. *Jpn. J. Clin. Oncol.* 32:162–166.

58. Freeman, B. A., P. R. Baker, …, M. d'Ischia. 2008. Nitro-fatty acid formation and signaling. *J. Biol. Chem.* 283:15515–15519.

59. Jain, K., A. Siddam, …, M. Balazy. 2008. The mechanism of oleic acid nitration by *NO(2). *Free Radic. Biol. Med.* 45:269–283.

Biophysical Journal, Volume *120*

Supplemental information

Identification of a critical lipid ratio in raft-like phases exposed to nitric oxide: An AFM study

Sanjai Karanth, Amir Azinfar, Christiane A. Helm, and Mihaela Delcea

Supplementary Information

Identification of a critical lipid ratio in raft-like phases exposed to nitric oxide: An AFM study

Sanjai Karanth[1, 2], Amir Azinfar[3], Christiane A. Helm[3] and Mihaela Delcea[1, 2, 4*]

[1]Institute of Biochemistry, University of Greifswald, Felix-Hausdorff-Straße 4, 17489 Greifswald, Germany;
[2]ZIK-HIKE, Zentrum für Innovationskompetenz "Humorale Immunreaktionen bei kardiovaskulären Erkrankungen", Fleischmannstraße 42, 17489 Greifswald, Germany;
[3]Institute of Physics, University of Greifswald, Felix-Hausdorff-Straße 6, 17489 Greifswald, Germany;
[4]DZHK (Deutsches Zentrum für Herz-Kreislauf-Forschung), partnersite Greifswald, Germany.
**Corresponding author: delceam@uni-greifswald.de*

Figure S1: Force histogram of tip-mica adhesive force to determine the non-specific interactions.

Figure S2: Break-through of raft-like phases with different SM and NO concentrations.

Figure S3. Lipid peroxidation assay to detect formation of MDA using N-palmitoyl-D-erythro-sphingosylphosphorylcholine (100% 16:0 SM, Avanti Polar Lipids, AL, USA).

Figure S4: Pull-out force histogram, AFM images and height profiles of POPC:POPS bilayer treated with nitric oxide.

Figure S5: Pull-out force histogram, AFM images and height profiles of DMPC:DMPG bilayer treated with nitric oxide.

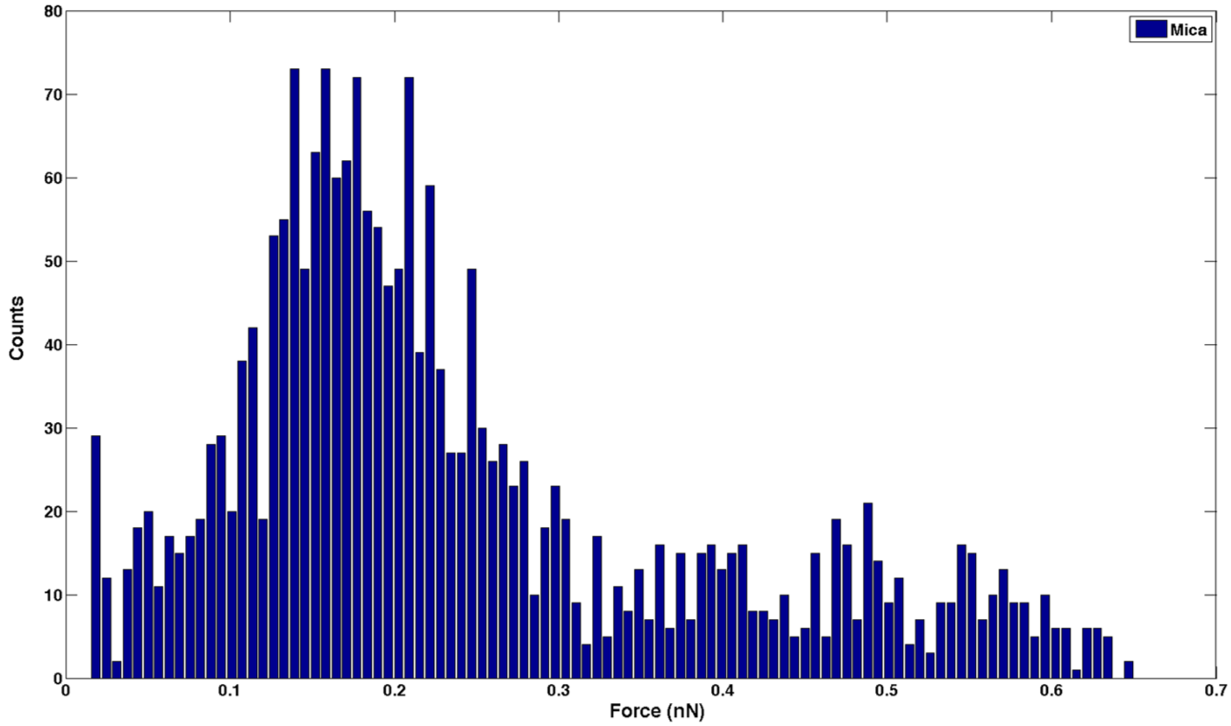

Figure S1: Histogram of pull-out forces between tip and mica surface to determine the non-specific attractive interactions. The histogram displayed forces ranging between 0.02 - 0.65 nN with maximum forces at around 0.2 nN.

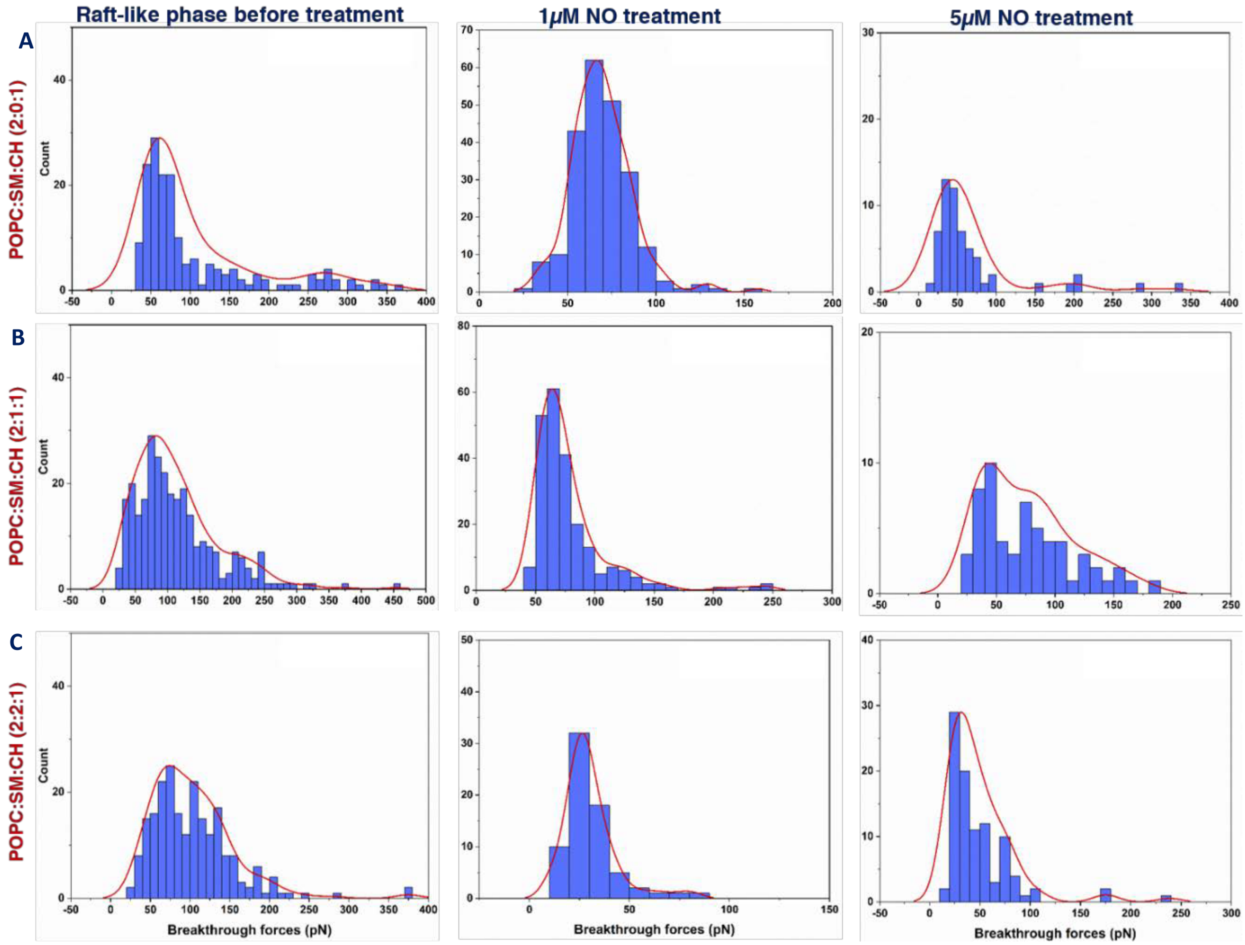

Figure S2: Histograms of break-through forces of raft-like phases (R_LP) with different SM concentration and NO treatment. Control samples *(left column)* showed break-through forces with a large distribution ranging between 25 pN - 400 pN. However, with increase in SM concentration, the distribution of break-through forces became more concentrated with half-width maxima of the Gaussian fit getting broader with SM addition. The peak break-through forces for 2:0:1 was found to be 68 pN *(A, left)* which increased to 80 pN *(B, left)* for the 2:1:1 ratio. Break-through forces were observed at 80 pN and 125 pN *(C, left)*, at almost equal intensities for 2:2:1 ratio. With addition of NO, the break-through forces and their distribution reduced for the 2:0:1 ratio and ranged from 25-150 pN *(A, middle)* without significant change in the peak force. This behavior was similar for 1μM NO (peak force=68 pN) and 5μM NO (peak force=50 pN) concentration. The 2:1:1 ratio showed slight reduction in the peak break-through forces from 80 pN to 75 pN at 1μM NO and 48 pN and 80 pN for 5 μM NO *(B, middle and right)*. A constant breakthrough force of 80 pN was detected throughout. However, the distribution seemed affected regardless of NO concentration with the force range changing from 25 pN -400 pN (control) to 25 pN- 250pN. The 2:2:1 ratio showed drastic changes in the break-through forces with peak force reduced to 27 pN at 1μM NO and 32.5 pN for 5μM NO *(C, middle and right)*. The force distribution was reduced and ranged between 25-100 pN for both NO concentrations. The above obtained forces correlated well with the pull-out forces (Figure 3 in the manuscript) giving a comprehensive understanding on the change in membrane permeability due to SM. Fit = KDE function.

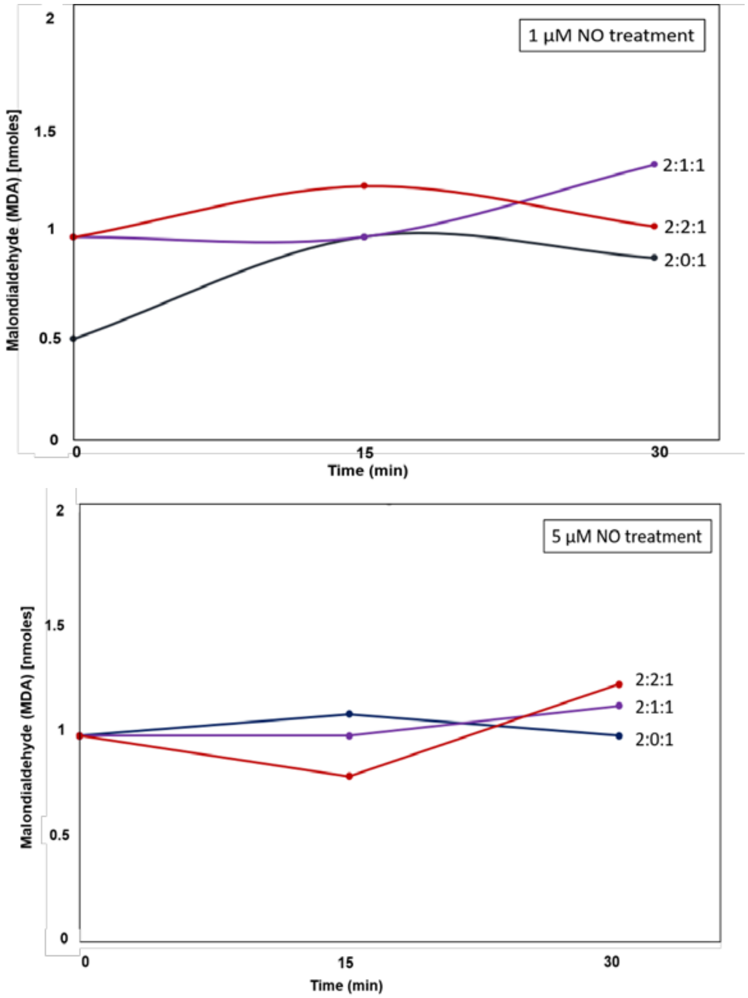

Figure S3: Lipid peroxidation assay to detect formation of MDA using N-palmitoyl-D-erythro-sphingosylphosphorylcholine (100% 16:0 SM) for different ratios. In Figure 6, we found that, increase in egg SM addition caused increase in MDA concentration. To confirm SM contribution in lipid peroxidation, egg SM was replaced with 100% 16:0 SM. The data presented here shows changes only due to POPC modifications the concentration of MDA detected without significant changes. Each data point is the average of three measurements.

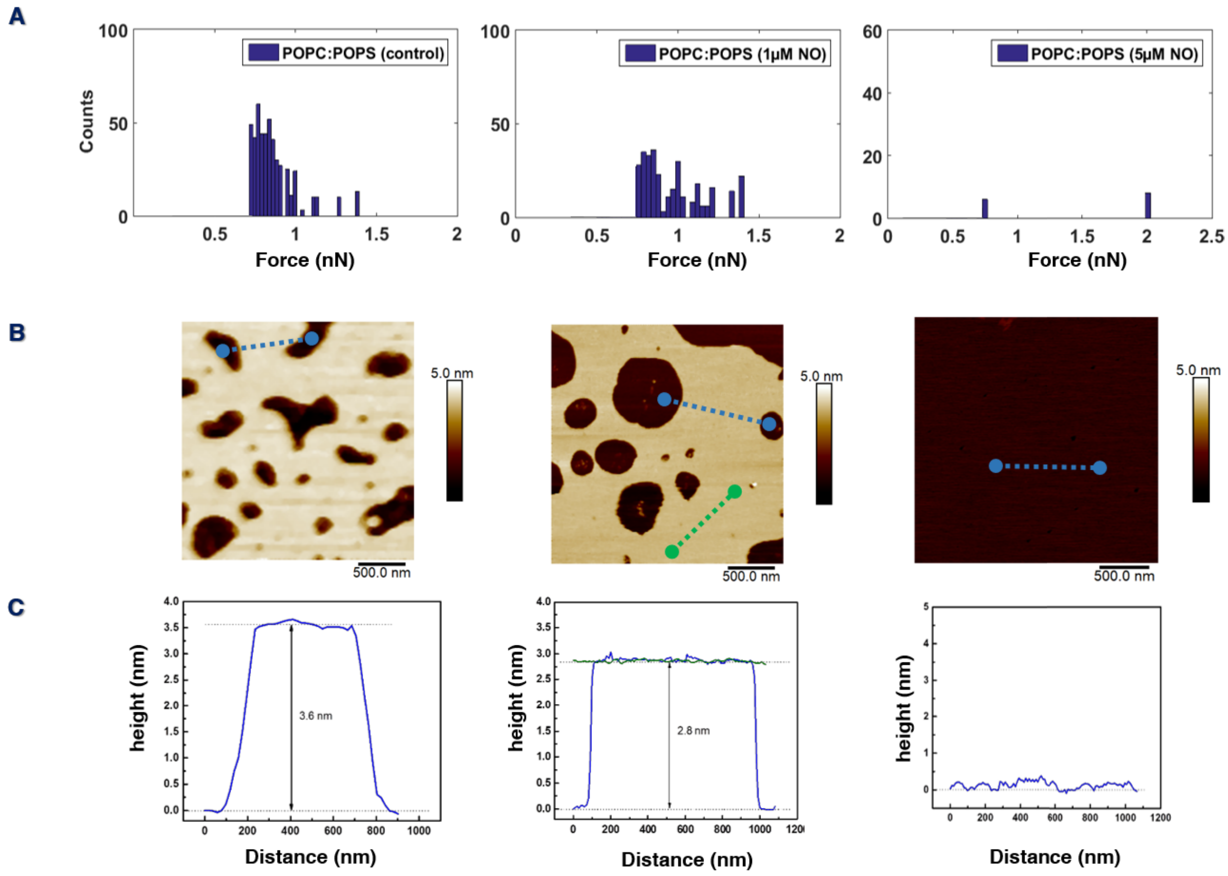

Figure S4: Pull-out force histogram (A), AFM images (B) and their corresponding height profiles (C) of POPC:POPS bilayer treated with 1 µM NO (*middle*) and 5 µM NO (*right*). POPC:POPS bilayer shows linear reduction in height of bilayer with increasing NO concentration. At 1 µM NO treatment, the maximum forces were similar to control (A, *left*), but complete membrane disruption was observed at 5 µM NO (A, *right*).

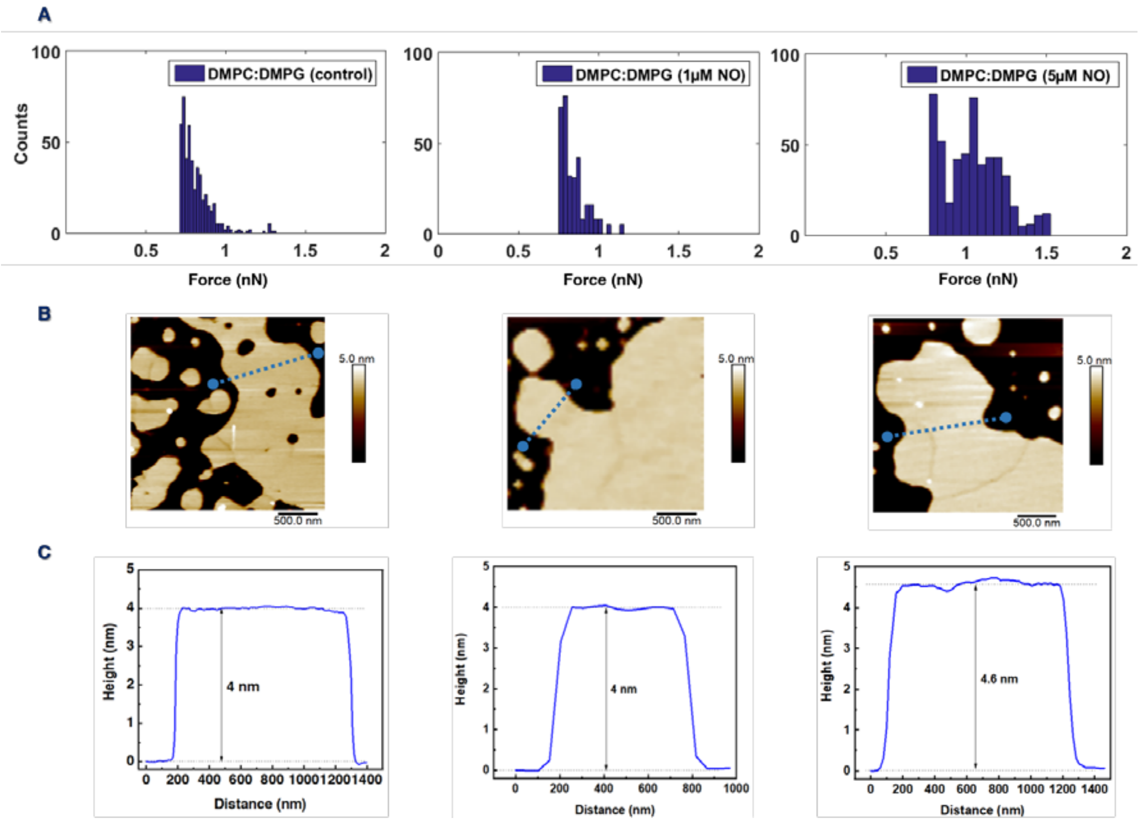

Figure S5: Pull-out force histogram (A), AFM images (B) and height profiles (C) of DMPC:DMPG bilayer treated with 1 µM NO (middle) and 5 µM NO (right). No changes in height or peak forces for 1 µM NO concentrations were found. At 5 µM NO, the maximum forces increased with increase in bilayer height, indicating NO dependent changes on saturated membranes.
